# Salicylic Acid Alleviates Aluminum Toxicity in Soybean Roots through Modulation of Reactive Oxygen Species Metabolism

**DOI:** 10.3389/fchem.2017.00096

**Published:** 2017-11-07

**Authors:** Ning Liu, Fengbin Song, Xiancan Zhu, Jiangfeng You, Zhenming Yang, Xiangnan Li

**Affiliations:** ^1^Northeast Institute of Geography and Agroecology, Chinese Academy of Sciences, Changchun, China; ^2^Institute of Special Animal and Plant Sciences, Chinese Academy of Agricultural Sciences, Changchun, China; ^3^Agriculture Ecology and Environment Laboratory, College of Plant Science, Jilin University, Changchun, China

**Keywords:** aluminum, *Glycine max* L., salicylic acid, hydrogen peroxide, paclobutrazol

## Abstract

As an important signal molecule, salicylic acid (SA) improves plant tolerance to aluminum (Al) stress. The objective of this study was to investigate the effects of exogenous SA application on the dynamics of endogenous SA and reactive oxygen species in soybean (*Glycine max* L.) exposed to Al stress. The roots of soybean seedlings were exposed to a combination of AlCl_3_ (30 μM) and SA (10 μM)/PAC (100 μM, paclobutrazol, SA biosynthesis inhibitor) for 3, 6, 9, and 12 h. Al stress induced an increase in endogenous SA concentration in a time-dependent manner, also verified by the up-regulated expression of *GmNPR1*, an SA-responsive gene. Al stress increased the activities of phenylalanine ammonia-lyase (PAL) and benzoic acid 2-hydroxylase (BA2H), and the contents of SA, ${\text{O}}_{2}^{-}$ and malondialdehyde (MDA) in the root apex. The application of exogenous SA increased PAL and BA2H, and reduced ${\text{O}}_{2}^{-}$ and MDA contents in soybean roots under Al stress. PAC inhibited the SA induced increase in BA2H activity. In addition, the SA application resulted in a rapid increase in hydrogen peroxide (H_2_O_2_) concentration under Al stress, followed by a sharp decrease. Compared with the plants exposed to Al alone, Al+SA plants possessed higher activities of superoxide dismutase, peroxidase, and ascorbate peroxidase, and lower catalase activity, indicating that SA alleviated Al-induced oxidative damage. These results suggested that PAL and BA2H were involved in Al-induced SA production and showed that SA alleviated the adverse effects of Al toxicity by modulating the cellular H_2_O_2_ level and the antioxidant enzyme activities in the soybean root apex.

## Introduction

Aluminum (Al) is the most abundant metal and the third most abundant element in the earth's crust after oxygen and silicon. Most of Al is incorporated into aluminosilicate soil minerals, with only small quantities appearing in soluble forms that can influence living organisms (May and Nordstrom, 1991). However, soil acidification leads to Al solution, which is continuing, at some locations all over the world (Guo et al., 2010). Al is released into the soil solution when the soil pH value <5 and becomes the most important factor limiting crop production on 67% of the total acid soil area (Eswaran et al., 1997). The most distinct symptom of Al toxicity is inhibition of root elongation, which involves cell wall structure, plasma membrane stability, inhibition of enzyme activity, and reactive oxygen species (ROS) homeostasis in roots (Barcelo and Poschenrieder, 2002). The Al-resistance mechanisms to deal with Al toxicity include Al exclusion and internal Al tolerance (Kochian et al., 2005). Al-exclusion mechanisms rely on the Al-activated secretion of organic compounds, such as, organic acid and phenolics, from the root apex (Kochian et al., 2005). Al-tolerance mechanisms involve detoxification of Al related to modification of the root cell wall structure and Al translocation from roots to shoots (Kochian et al., 2015).

The phytohormone salicylic acid (SA) is a small phenolic compound that functions as an important signaling molecule in regulating plant growth and development and inducing plant defense responses under biotic and abiotic stress, such as, salinity, drought, and heavy metal toxicity (Horváth et al., 2007; Rivas-San and Plasencia, 2011; Sinha et al., 2015). Pathogen infection induces SA biosynthesis at infection sites, which regulates the expression of several defense genes, including pathogenesis-related (*PR*) genes and non-expresser of pathogenesis-related (*NPR*) genes, leading to systemic acquired resistance (SAR) (Mou et al., 2003; Durrant and Dong, 2004). It has been shown that *Arabidopsis NPR1* plays a role in SA-mediated antioxidant defense under Al stress (Guo et al., 2014; Zhang et al., 2014). The protective effects of exogenous SA on Al-toxicity by preventing Al-induced oxidative stress have been extensively described in various plant species, such as, tomato (Surapu et al., 2014), *Coffea arabica* (Mu-oz-Sanchez et al., 2013), *Cassia tora* (Yang et al., 2003), cauliflower (Sinha et al., 2015), barley (Song et al., 2011), wheat (Uysal et al., 2009), *Matricaria chamomilla* (Kováčik et al., 2012), and rice (Pandey et al., 2013). It was found that SA suppressed the Al-induced enhancement of superoxide dismutase (SOD), glutathione peroxidase (GPX) and ascorbate peroxidase (APX) activities and increased catalase (CAT) activity in rice plants exposed to Al stress (Pandey et al., 2013). In addition, SA alleviates Al toxicity in *C. tora* by significantly enhancing peroxidase (POD) activity (Yang et al., 2003). This suggests that exogenous SA is involved in mitigating Al toxicity, and the SA' effects vary with plant species and experimental conditions.

SA is synthesized by two pathways, the isochorismate pathway and the phenylalanine ammonia lyase (PAL) pathway (Chen et al., 2009; Vlot et al., 2009). In plants, SA is mainly synthesized through the phenylalanine route localized in the cytoplasm. In this pathway, PAL and benzoic-acid-2-hydroylase (BA2H) are the key enzymes in SA biosynthesis (Chen et al., 2009). Dong et al. (2014) reported that chilling stimulates the enzyme activities and the expression of *PAL* and *BA2H* genes, that in turn induces the moderate accumulation of endogenous SA in cucumber seedlings, indicating that the PAL pathway contributes to chilling-induced SA production.

The objective of this study was to examine the effect of exogenous SA and of SA synthesis inhibitor paclobutrazol (PAC) on root elongation rate, and Al content, SA synthesis, membrane injury, and antioxidant system in soybean roots. We hypothesized that (1) exogenous SA would increase activities of PAL and BA2H and endogenous SA synthesis, thus alleviating the negative effect of Al stress on membrane injury and antioxidant system; (2) The effects of exogenous SA would be limited by adding the exogenous PAC, which act as inhibitor of endogenous SA synthesis.

## Materials and methods

### Plant materials and treatments

Soybean (*Glycine max* L.cv. Jiyu70) seeds were surface sterilized for 5 min in 1.0% (v/v) NaClO, rinsed thoroughly with tap water and germinated in peat-moss for 3 days at 25°C in the dark. The seedlings were transferred to plastic containers filled with 1.0 L nutrient solution (pH 4.5) (Horst et al., 1992). The medium was refreshed every 3 days. The climate conditions in the chamber were set as follows: day/night temperature 25/22°C, photoperiod 14 h, light intensity 300 μmol photons m^−2^ s^−1^ (sodium lamp Philips SON, 400 W), and relative humidity 70%.

After 7 days growth in the nutrient solution, soybean seedlings were transferred to 0.5 mM CaCl_2_ solution (pH 4.5) for 12 h in the dark. Then, the seedlings were subjected to different treatment solutions. In the time course experiment, the roots of seedlings were exposed to 0.5 mM CaCl_2_ solution (pH 4.5) containing 0 or 30 μM AlCl_3_ with or without 10 μM SA for 3, 6, 9, or 12 h. In the pharmacological experiment, the roots of seedlings were subjected to 0.5 mM CaCl_2_ solution (pH 4.5) containing 0 or 30 μM AlCl_3_ with or without 10 μM SA or 100 μM PAC. Each experiment was repeated at least three times. Root apices (0–3 cm) were excised and stored in a −80°C freezer until the determination of the SA content and an enzyme activity assay.

### SA content determination

The total SA content (free plus bound) in fresh root tips was determined according to the method used by Liu et al. (2012). The soybean roots (about 1.0 g) were ground in liquid nitrogen, 4 mL of 5% trichloroacetic acid (TCA), 16 mL of double distilled H_2_O and 30 mL of ethyl ether were added to the homogenate and transferred to 50 mL centrifuge tube, fully shaken, and extracted for 12 h. After 5 min centrifugation at 10,000 g, the ethyl ether supernatants was partitioned, the aqueous phase was extracted two times in succession with ethyl ether. Three combined extracts were condensed by rotary evaporation at 40°C and the residue was dissolved in 1 mL methanol and acetate buffer (pH 3.2) (1:1, v/v) for free-SA analysis. Bound SA content was indirectly quantified by acid hydrolysis of the compounds remaining in the aqueous phase after organic extraction. Acid hydrolysis was performed by adding 18.5% HCl to the aqueous phase in order to make the final concentration 3.2%. The samples were incubated at 80°C in a water bath for about 1 h. After cooling, they were extracted with ethyl ether for three times. The combined ethyl ether phase supernatants were condensed by rotary evaporation at 40°C and 1 mL of the mixture of methanol and acetate buffer (pH 3.2) (1:1, v/v) was added to 50 ml rotary evaporator flask, and dissolved fraction was transferred to 1.5 mL Eppendorf vials containing the bound SA. Quantitative analysis of free and bound SA were performed by high-performance liquid chromatography (HPLC, LC 20 AT, Shimadzu, Tokyo, Japan) equipped with a reverse-phase C18 column (VP-ODS, 150 mm × 4.6 mm) and a fluorescence detector (excitation wavelength = 310, emission wavelength = 415 nm). All the samples were filtered through 0.3 μm microporous filters before injection.

### Activities of PAL and BA2H assay

Root apices (0–3 cm) were excised for the PAL activity assay, following Ferrarese et al. (2000) with some modifications. Samples (1.0 g) were ground at 4°C in 0.1 M sodium borate buffer (pH 8.8), containing 15 mM β-mercaptoethanol, 5 mM EDTA, 1 mM phenylmethanesulfonyl fluoride, and 0.15% polyvinyl pyrrolidone (w/v). Homogenates were centrifuged (15,000 g, 4°C, 20 min), and the supernatant was used for enzyme preparation. The reaction mixture contained 0.1 M of borate buffer (pH 8.8), 15 mM L-phenylalanine and 1 mL enzyme extract in a 5 mL volume. The reaction proceeded for 1 h at 37°C and was interrupted by the addition of 0.5 mL 6 M HCl. The activity of PAL was determined spectrophotometrically at a wavelength of 290 nm.

BA2H activity was determined using the method presented by Qian et al. (2009). Samples (1.0 g) were frozen in liquid nitrogen and ground in a chilled mortar. The frozen powder was added to 0.03 M potassium phosphate buffer (pH 7.5) containing 1 mM EDTA, 1 mM sodium benzoate and 0.5 mM dithiothreitol. The homogenate was centrifuged at 12,000 g for 15 min at 4°C. The supernatant was used for determination of BA2H enzyme activity. The reaction mixture (final volume of 4 mL) contained 1.2 mL 1 M Tri-HCl buffer at pH 7.5, 0.4 mL H_2_O, 0.6 mL 150 μM NADPH, 0.6 mL 20 μM FAD, and 1.2 mL enzyme extract. The reaction mixture was partitioned into two parts. One part was added to 1 mL 1 mM benzoic acid to start the enzymatic reaction; the other part was added to 1 mL H_2_O. The product of the reaction catalyzed by NADPH was determined spectrophotometrically at 340 nm. Protein content was determined according to the method of Bradford (1976), using bovine serum albumin as a standard.

### ROS assay

${\text{O}}_{2}^{-}$ production was determined according to the method of Shi and Zhu (2008).

The malondialdehyde (MDA) content was measured as described by Heath and Packer (1968). Briefly, root tips (1.0 g) were ground in 3 mL 0.1% trichloroacetic acid (TCA) solution. The homogenate was centrifuged at 15,000 g for 10 min, and 0.5 mL the supernatant was assayed for MDA content.

The H_2_O_2_ concentration in crude extracts from soybean roots was measured by spectrofluorometry, according to the method used by Jana and Choudhuri (1982), and the absorbance of the titanium-peroxide complex was monitored at 410 nm. Using a standard calibration plot, the A_410_ readings were converted to corresponding H_2_O_2_ concentrations.

For H_2_O_2_ detection by fluorescence microscopy according to Rodríguez-Serrano et al. (2009). Soybean root tips (0–1 cm) were excised in centrifuge tube (1.5 mL). The 2′,7′-dichlorofluorescin diacetate (DCF-DA, 25 μM) was used as a fluorescent probe to stain the root tips for 20 min at 37°C in the dark. Then, the root tips were washed thrice with Tris-HCl (pH 7.2) buffer. The stained root tips were observed under a Nikon Eclipse 80i upright fluorescence microscope equipped with a mercury lamp (100 W) and a C-FL-C 30 mm Epi-Fluorescence Filter Block (MBE44725, FITC) consisting of excitation filter EX465-495, Dichroic Mirror DM505, Barrier filter BA512-558. The excitation and emission wavelengths of H2DCF-DA are 485 and 530 nm. The CCD camera (Nikon Fi2) and the NIS-Elements F software were used to capture the images.

### Antioxidative enzyme activity assays

Soybean root tips (1.0 g) were frozen in liquid nitrogen and homogenized in 2 mL 50 mM sodium phosphate buffer (pH 7.8) containing 0.1 mM EDTA and 1% (w/v) PVP. The homogenate was centrifuged at 15,000 g for 20 min at 4°C, and the supernatant was used for the following enzyme assays.

The activities of SOD, CAT, and APX were determined as described by Jiang and Zhang (2001). Total SOD activity was assayed by monitoring the inhibition of photochemical reduction of nitro blue tetrazolium (NBT). One unit of SOD activity was defined as the amount of enzyme required to cause 50% inhibition of the reduction of NBT as monitored at 560 nm. CAT activity was determined by measuring the rate of decomposition of H_2_O_2_ at 240 nm for 5 min. The enzyme activity was calculated using an extinction coefficient of 39.4 mM^−1^ cm^−1^. APX activity was determined by measuring the decrease rate of the H_2_O_2_-dependent oxidation of ascorbate at 290 nm for 5 min. The enzyme activity was calculated using an extinction coefficient of 2.8 mM^−1^ cm^−1^. Peroxidase (POD) activity was monitored by oxidation of guaiacol using H_2_O_2_. The enzyme activity was calculated using an extinction coefficient of 26.6 mM^−1^ cm^−1^ at 470 nm.

### Total RNA extraction and gene expression analysis

Total RNA was isolated using RNA isoreagent (TaKaRa Bio Inc., Shinga, Japan) according to the manufacturer's protocol. cDNA was synthesized from 1 μg total RNA in a 10 μL aliquot using Moloney Murine Leukemia Virus reverse transcriptase (M-MLV, TaKaRa Bio Inc.) at 42°C for 1 h with the oligo-dTadaptor primer (TaKaRa Bio Inc.) following the manufacturer's protocol. Real-time quantitative PCR was performed using 0.5 μL of reverse transcription production in a 25-μL reaction volume with SYBR Premix Ex Tag (TaKaRa Bio Inc.) on an ABIPRISM 7500 Sequence Detection System (Applied Biosystems, Foster City, CA, USA). Real-time PCR was performed using gene-specific primers: β-tubulin was used as a house-keeping gene according to Xu et al. (2010), and the GmNPR1 Gene-Bank accession number is FJ418595; upstream primer 5′-ATGGCAAGGTTGGATATGAAGC-3′ and downstream primer 5′-TGGCAGGTCTACACGCATCA-3′; the amplification size of the product was 132 bp. Each sample was run in triplicate. The relative transcript abundance was calculated using the 2^−ΔΔCT^ method.

### Statistical analysis

All data were statistically analyzed using SPSS 13.0 software (SPSS Inc., Chicago, IL, USA), including variance analysis and multiple mean comparison using Tukey's test. The figures were drawn using Kyplot software (OriginLab Corporation, Northampton, MA, USA).

## Results

### Effect of SA and PAC on root elongation and Al content

**Figure 1:**
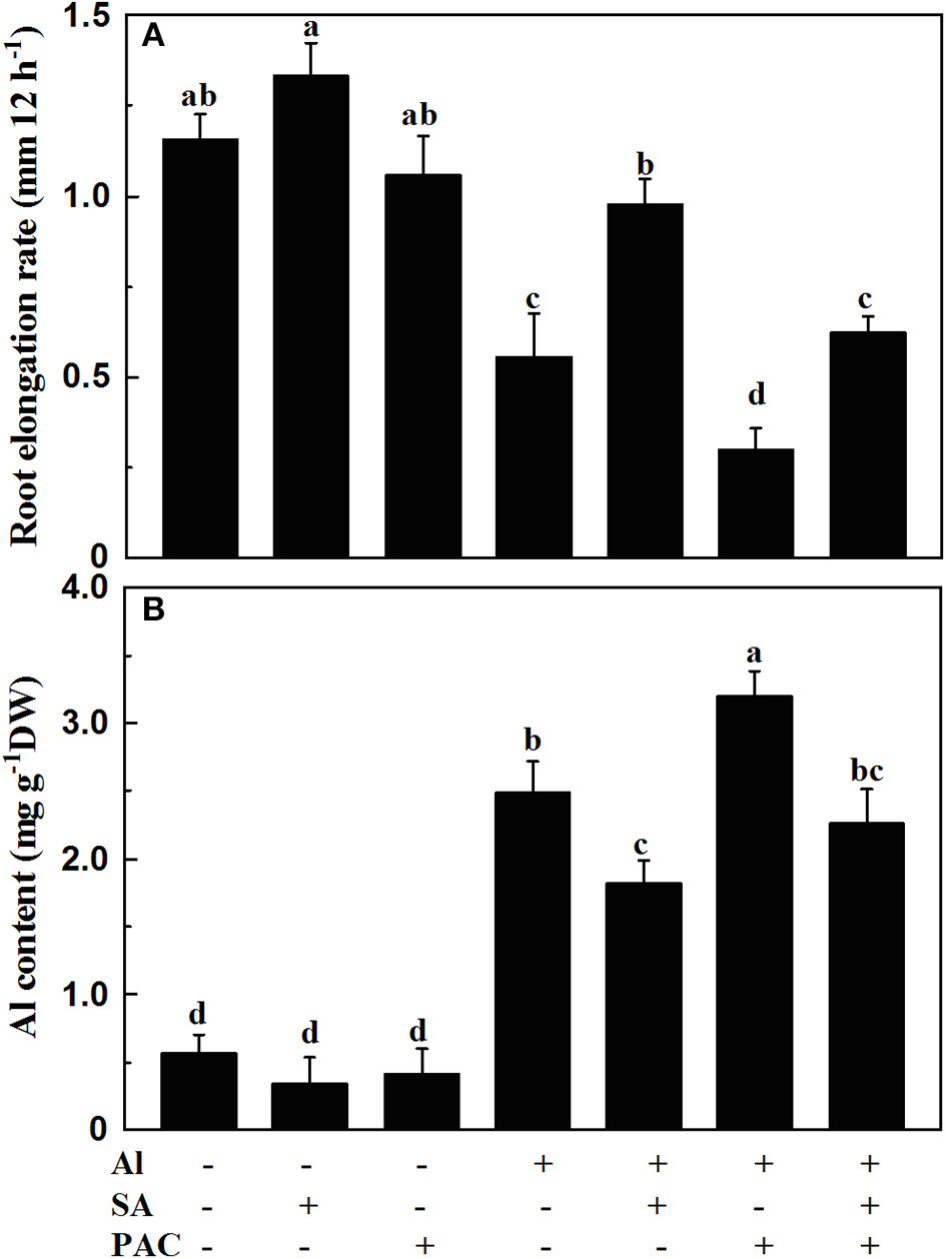
Root elongation rate **(A)** and Al content **(B)** of 1-cm root tips of soybean. Four-day-old seedlings were exposed to 0.5 mM CaCl_2_ solution (pH 4.5) containing 10 μM SA and 100 μM PAC with or without 30 μM AlCl_3_ for 12 h. Bars represent means ± SD, *n* = 15 for **(A)** and *n* = 9 for **(B)**. Significant differences among the treatments are indicated by different lower case letters (*p* < 0.05, Tukey's test).

The root elongation rate was reduced by 51.7% after 12 h' Al treatment (Figure 1A). The root elongation rate only decreased by 15.5% when roots were treated with the solution containing both Al and 10 μM SA. This showed that SA alleviated Al-induced inhibition of root elongation.

The alleviating effect of SA on Al-induced inhibition of root elongation suggested that the inhibition of root elongation by Al may be caused by the reductions in endogenous SA concentration in soybean roots. To test this hypothesis, the SA biosynthesis inhibitor PAC was used in the absence and presence of Al and SA. Treatment with 100 μM PAC for 12 h aggravated Al-induced root elongation inhibition, which could be reversed by exogenous SA supply (Figure 1A). In addition, SA decreased, while PAC increased Al content in the root tips of the soybean seedlings under Al stress (Figure 1B).

### Endogenous SA production in root tips under Al stress

**Figure 2:**
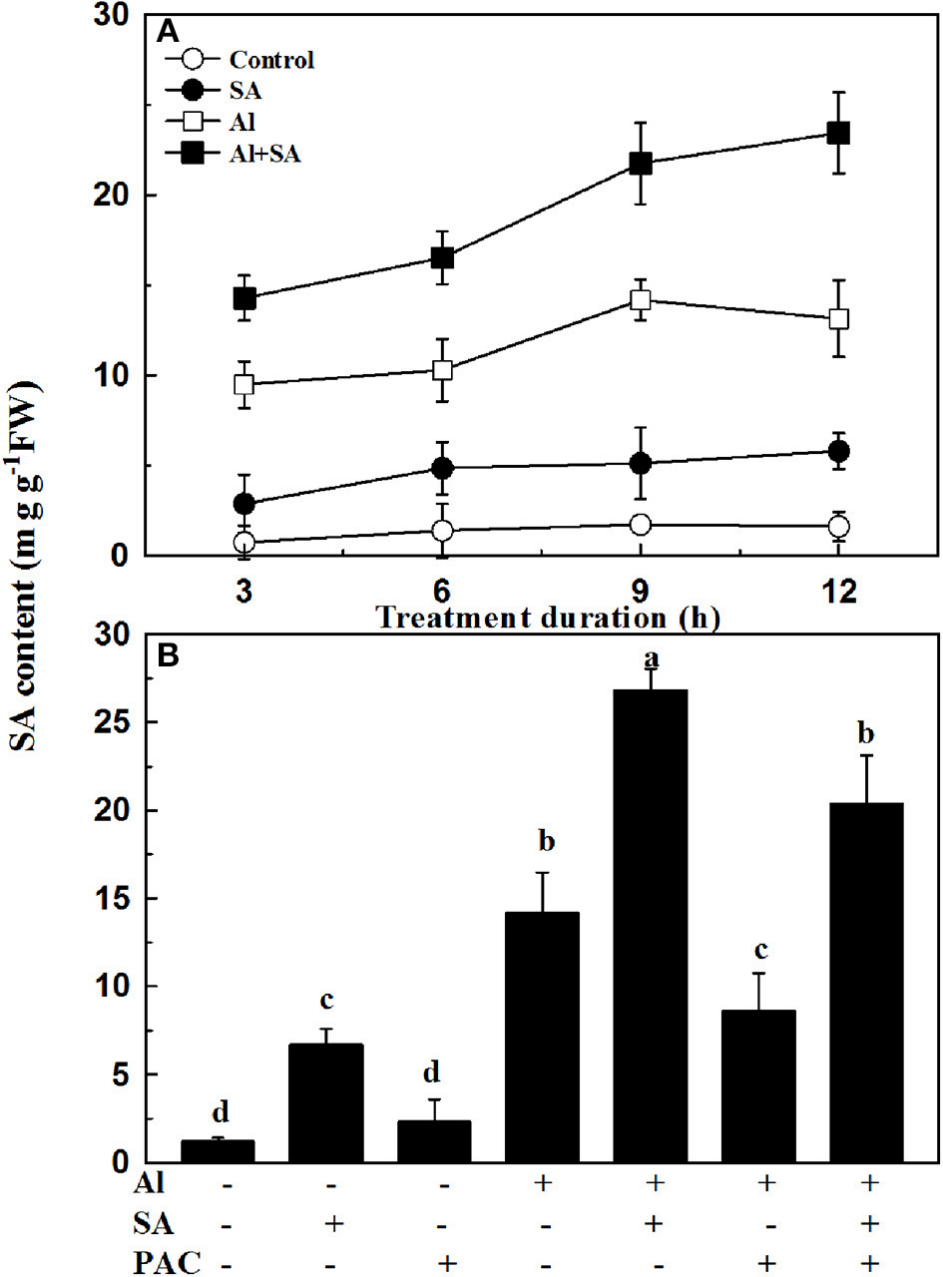
Endogenous SA production in the root tips of soybean seedlings. **(A)** Seven-day-old seedlings were exposed to 0 or 30 μM AlCl_3_ with or without 10 μM SA in 0.5 mM CaCl_2_ solution (pH 4.5) for 3, 6, 9, and 12 h. Then, root tips (0–3 cm) were excised and analyzed to determine the SA content. **(B)** Seven-day-old seedlings were subjected to AlCl_3_ (0, 30 μM), SA (0, 10 μM), and PAC (0, 100 μM) in 0.5 mM CaCl_2_ solution (pH 4.5) for 12 h. Root tips (3 cm) were cut, and the SA content was determined. Bars represent means ± SD of three individual replicates. Significant differences among the treatments are indicated by different lower case letters (*p* < 0.05, Tukey's test).

To confirm whether Al-induced inhibition of root elongation was related to endogenous SA concentration, the concentrations of total SA were investigated at 3-h intervals over a period of 12 h (Figure 2A). The effect of SA on Al-induced endogenous SA concentrations in soybean roots was also examined under PAC treatment (Figure 2B). In the presence of Al, both SA and PAC caused higher SA concentration as compared to the corresponding controls without Al. Exogenous SA markedly enhanced the Al-induced endogenous SA concentrations by 88.7% compared with the Al treatment without SA. However, the application of PAC decreased the SA content regardless of SA application.

### Source of endogenous SA under Al stress

**Figure 3:**
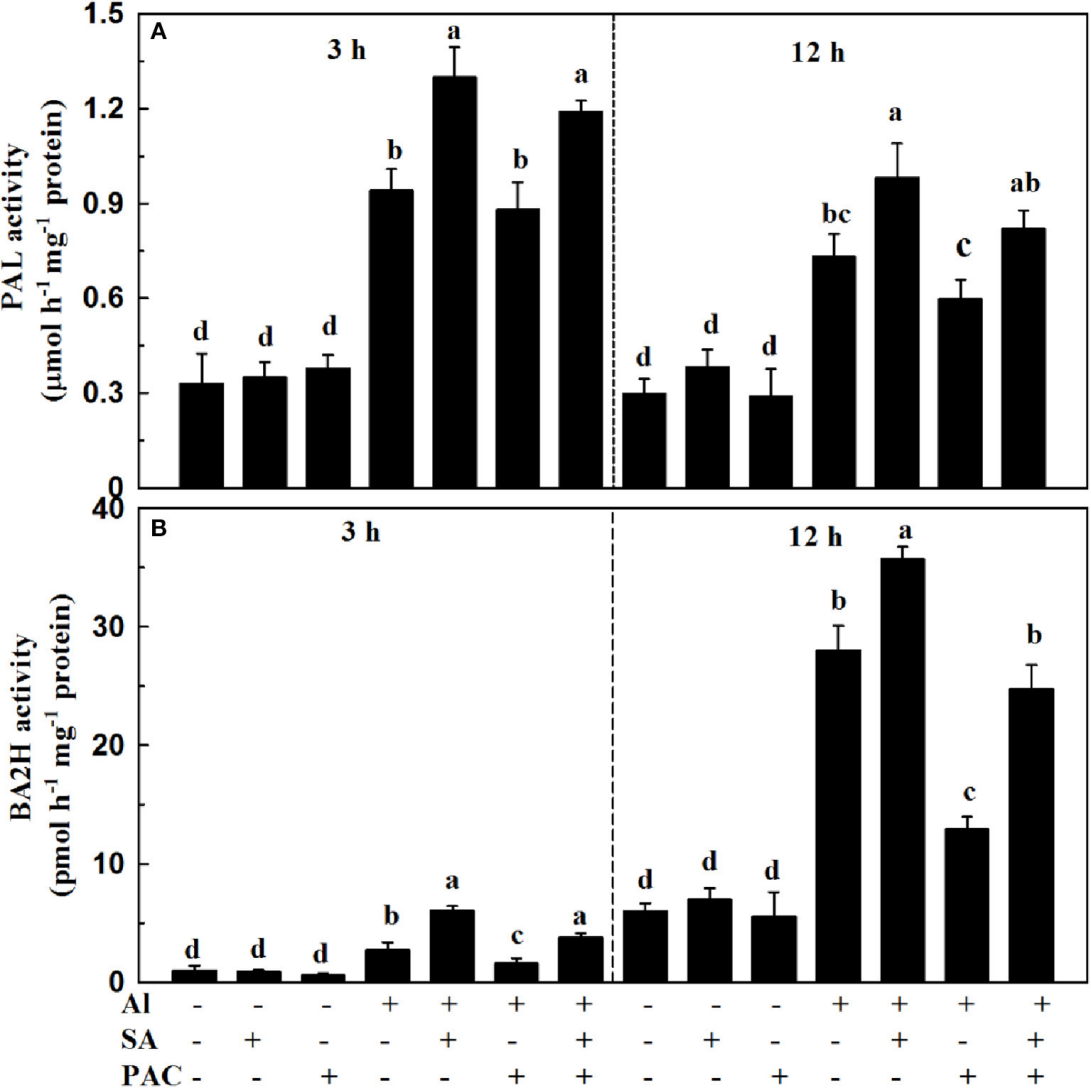
Activities of PAL **(A)** and BA2H **(B)** in root apices of soybean under Al stress. Seven-day-old seedlings were exposed to AlCl_3_ (0, 30 μM), SA (0, 10 μM), and PAC (0, 100 μM) in 0.5 mM CaCl_2_ solution (pH 4.5) for 3 and 12 h. Root tips (3 cm) were collected to determine the activities of PAL and BA2H. Bars represent means ± SD of three individual replicates. Significant differences among the treatments are indicated by different lower case letters (*p* < 0.05, Tukey's test).

PAL and BA2H serve as two key enzymes responsible for SA biosynthesis in plants. To determine the contribution of PAL and BA2H enzymatic pathways to SA production under Al stress, we analyzed the activities of the two enzymes at 3 and 12 h. As shown in Figure 3, the PAL activity was increased after 3 h of Al treatment, while it was decreased slightly at 12 h. Al stress elevated the activity of BA2H at 3 and 12 h, i.e., by 2.8-fold and 4.7-fold, respectively, in relation to the control. Application of exogenous SA under Al stress further enhanced the PAL and BA2H activities at 3 and 12 h. It has been reported that PAC is an effective inhibitor of BA2H during SA biosynthesis. As expected, the activity of BA2H, but not PAL, was markedly inhibited in the PAC treatment under Al stress.

### Effect of SA on the expression of the *GmNPR1* gene under Al stress

**Figure 4:**
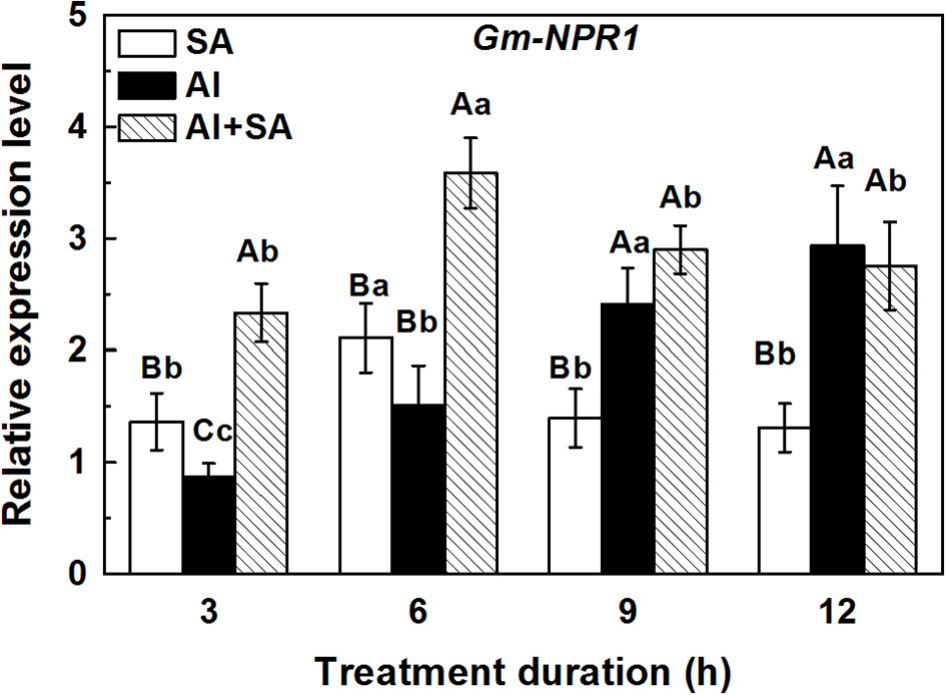
Relative expression level of the *GmNPR1* gene in 1-cm root tips of soybean. Seven-day-old seedlings were transferred to 0.5 mM CaCl_2_ solution (pH 4.5) containing AlCl_3_ (0, 30 μM) and SA (0, 10 μM) for 3, 6, 9, and 12 h. Total RNA was extracted from eight primary root tips. The expression levels of RNA were measured using qRT-PCR. For normalization of gene expression, the constitutively expressed β*-tubulin* gene of soybean was used as a reference gene. Significant differences among the times and treatments are indicated by different upper and lower case letters respectively (*p* < 0.05, Tukey's test).

To test SA accumulation under Al stress at the transcriptional level, we examined the expression of *GmNPR1* at 3, 6, 9, and 12 h (Figure 4). In soybean roots, the transcriptional level of *GmNPR1* was strikingly increased by Al treatment with the elongation of treatment duration. This result was consistent with the endogenous SA accumulation pattern during Al treatment. In the SA plus Al treatment, the gene expression could be rapidly induced and reached a maximum at 6 h, which was over 2-fold higher than that in response to Al alone. However, the high expression of *GmNPR1* could not be maintained until 12 h.

### Effect of SA on ${\text{O}}_{2}^{-}$ and MDA accumulation under Al stress

**Figure 5:**
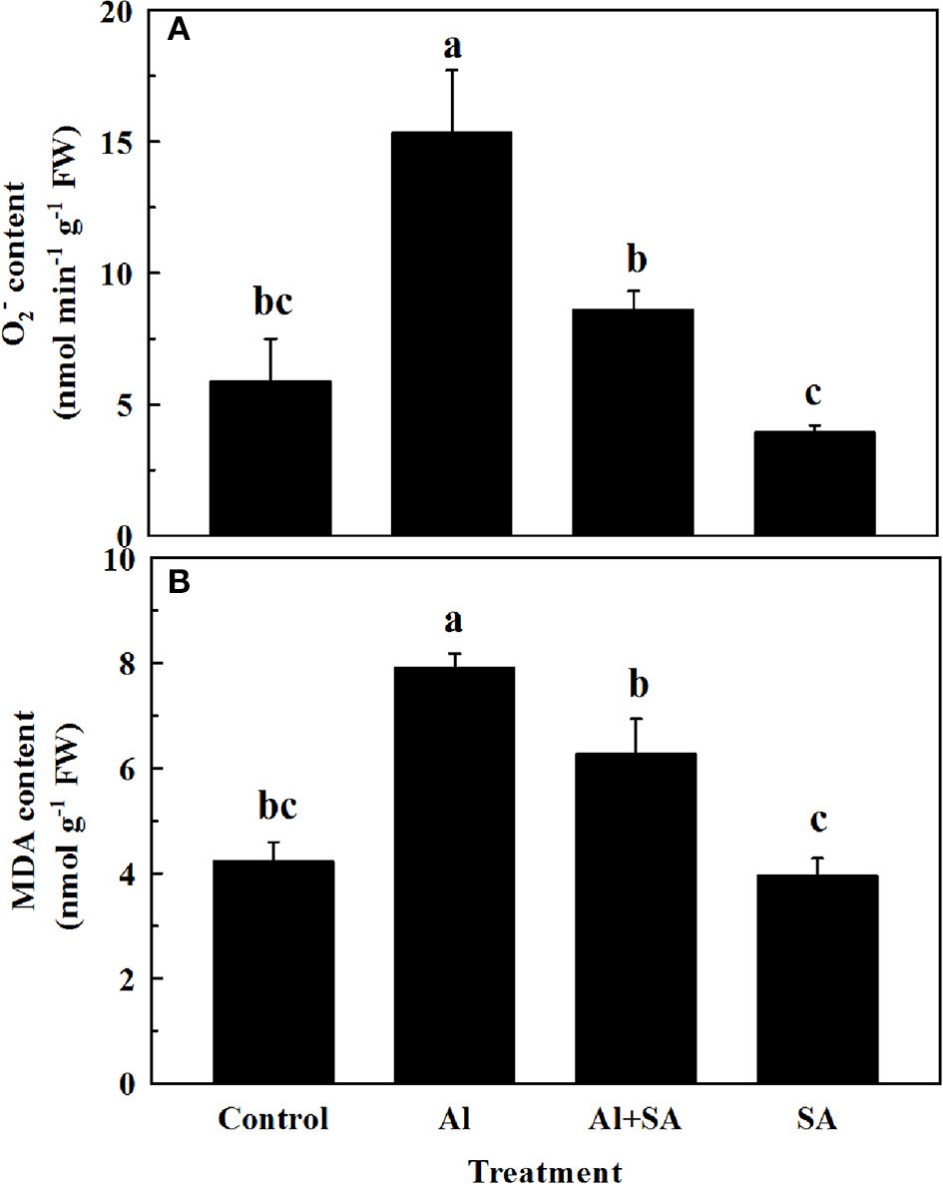
${\text{O}}_{2}^{-}$ **(A)** and MDA **(B)** content in root apices of soybean. Seven-day-old seedlings were exposed to 0.5 mM CaCl_2_ solution (pH 4.5) containing 0 or 30 AlCl_3_ with or without 10 μM SA for 12 h. Bars represent means ± SD of three individual replicates. Significant differences among the treatments are indicated by different lower case letters (*p* < 0.05, Tukey's test).

To assess the effect of SA on ROS and lipid peroxidation under Al stress, the accumulation of ${\text{O}}_{2}^{-}$ and MDA was detected in soybean roots after 12 h of Al treatment (Figure 5). Adding exogenous SA reduced the contents of ${\text{O}}_{2}^{-}$ and MDA by 44 and 20%, respectively, compared with Al treatment alone, which indicated SA mitigated oxidative damage caused by Al stress.

### Effect of SA on the H_2_O_2_ content and activity of antioxidant enzymes under Al stress

**Figure 6:**
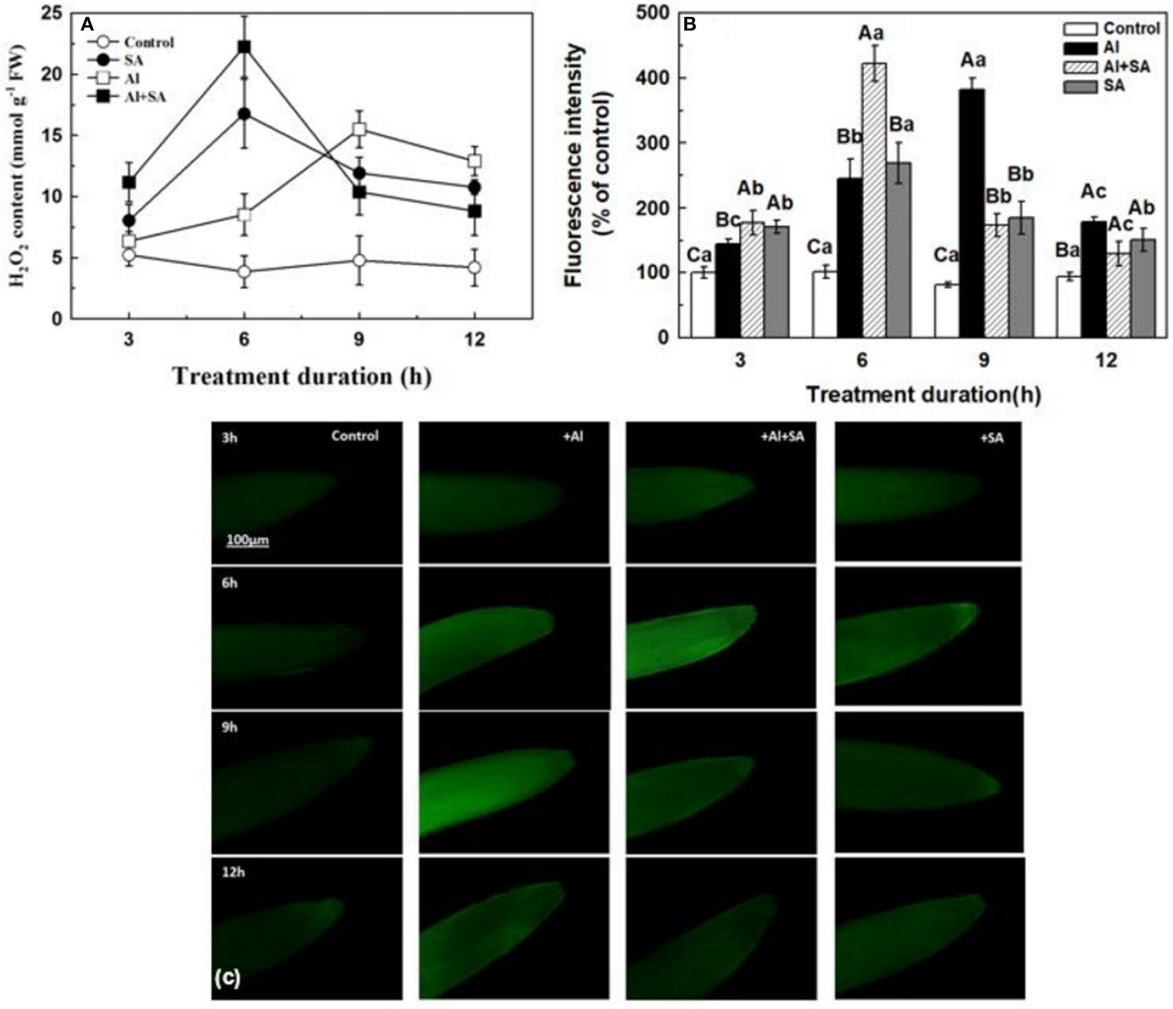
H_2_O_2_ content **(A)** and fluorescence intensity **(B)** in root apices of soybean. **(A)** Seven-day-old seedlings were treated with or without SA under Al stress in 0.5 mM CaCl_2_ solution (pH 4.5) for 3, 6, 9, and 12 h. The H_2_O_2_ content in the root tips of soybean seedlings was determined using spectrophotometry. Bars represent means ± SD of three individual replicates. **(C)** The endogenous H_2_O_2_ level was monitored by labeling H_2_O_2_ using DCF-DA. Root tips (0–1 cm) were excised and then incubated with 25 μM of DCF-DA in the dark for 20 min at 37°C, after which the root tips were rinsed two to three times using Tris-HCl (pH 7.2). The endogenous H_2_O_2_ content was measured using a Nikon fluorescence microscope (EX 485 nm/EM 530 nm). The fluorescence value was determined using a data acquisition system. Significant differences among the times and treatments are indicated by different upper and lower case letters respectively (*p* < 0.05, Tukey's test).

To determine how H_2_O_2_ changed in response to exogenous SA under Al stress, we detected H_2_O_2_ production using spectrophotometry (Figure 6A). Compared with the control, SA application significantly increased H_2_O_2_ content, and an oxidative burst occurred at the 6th h; Al application also increased H_2_O_2_ content, but the oxidative burst occurred at the 9th h (Figure 6A). Compared to only Al treatment, the concomitant application of Al and exogenous SA caused an oxidative burst at the 6th h, which was even stronger than with only SA; after that, the H_2_O_2_ content promptly declined to levels significantly lower than in the only Al-treated samples. To provide extra information of the H_2_O_2_ accumulation in different treatments, we used a H_2_O_2_-specific fluorescent probe, i.e., H_2_DCF-DA (Figures 6B,C), and the results were similar with spectrophotometry methods.

**Figure 7:**
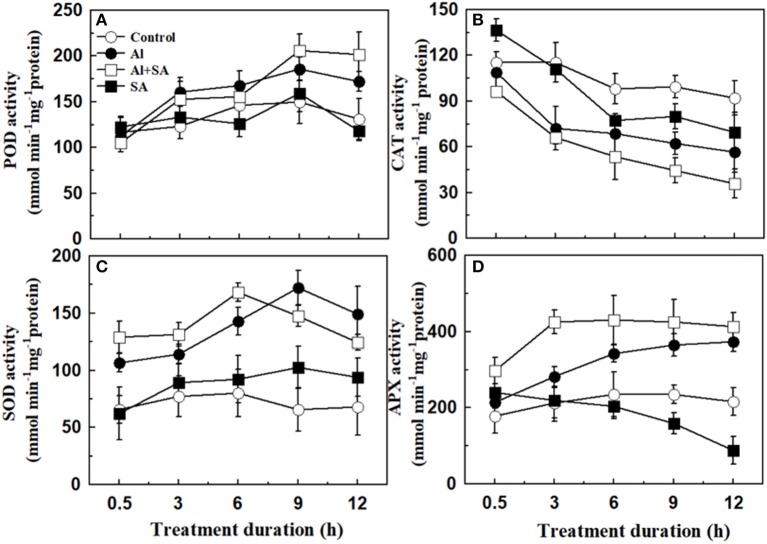
Activity of antioxidant enzymes in soybean seedlings. Seven-day-old seedlings were treated with 30 μM AlCl_3_ with or without 10 μM SA in 0.5 mM CaCl_2_ solution (pH 4.5) for 0.5, 3, 6, 9, and 12 h. The root tips (1 cm) were then collected to determine the activities of antioxidant enzymes: peroxidase (POD; **A**), catalase (CAT; **B**); superoxide dismutase (SOD, **C**) and ascorbate peroxidase (APX; **D**). Bars represent means ± SD of three individual replicates.

We also measured the activities of the antioxidant enzymes POD, CAT, SOD, and APX to analyze the source of H_2_O_2_ formation and protection against oxidative stress. As shown in Figure 7, Al-induced POD, SOD, and APX activities increased gradually with the elongation of treatment duration. SOD activity at 9 h was the highest and roughly paralleled the changes in the H_2_O_2_ content under Al stress. However, an Al-induced variation tendency in CAT activity occurred with different patterns under Al stress. CAT activity was repressed rapidly at 3 h and was then maintained at a relatively stable state until the end of the Al treatment. Application of exogenous SA further increased the activities of Al-induced POD and APX within 12 h; SOD activity peaked at 6 h, and CAT activity decreased 3 h after Al treatment.

## Discussion

The first hypothesis about exogenous SA effect was well supported by our results. It was found that exogenous Al caused obviously toxicity on root, i.e., it significantly decreased the root elongation rate and increased the Al content inside it; however, exogenous SA significantly ameliorated Al-induced inhibition of root elongation and reduced Al accumulation. Metal ion stress can enhance the SA level in a range of plant species (Metwally et al., 2003; Yao et al., 2012; Guan et al., 2015). The first hypothesis was also supported by the effects of exogenous SA on the accumulation of endogenous SA under both conditions, i.e., with and without Al stress. Additionally, exogenous SA continuously increased endogenous SA content throughout the 12-h experiment, which was consistent with the results shown in a study by Wang et al. (2004), who found that SA increased progressively up to 4-fold higher levels at the end of a 12 h exposure to Al in roots of *C. tora*. Exogenous SA had a synergistic and additive effect on endogenous SA under Al stress. These results indicated that changes in endogenous SA concentration in the root tips of soybean were closely associated with Al tolerance. Similar to the positive effects on endogenous SA content, exogenous SA also significantly increased PAL and BA2H activities, suggesting that endogenous SA content was strongly related to PAL and BA2H activities. Interestingly, without Al stress, exogenous SA did not show significant effects on ${\text{O}}_{2}^{-}$ and MDA contents, but significantly decreased their contents under Al stress. This may be because exogenous SA increased endogenous SA content under Al toxicity, then decreased the ${\text{O}}_{2}^{-}$ and MDA contents.

The second hypothesis was also well supported by our results, i.e., that the addition of exogenous PAC brought about opposite effects as compared to exogenous SA supply, especially under Al stress. In fact, the addition of the SA biosynthesis inhibitor PAC exacerbated the Al-induced damage, leading to lower root elongation rates accompanied by a higher root Al content, thus again confirming the alleviation effect of Al toxicity by SA and especially by exogenous SA. Accordingly, under Al stress, the addition of PAC reduced the ameliorating effects caused by the administration of exogenous SA, reducing the levels of endogenous SA contents and also the BA2H activity. This strongly suggested that PAC may impair endogenous SA synthesis by specifically inhibiting BA2H activity. Consistent with this, it has been shown that PAC pre-treatment resulted in the disappearance of a conjugated SA peak in heat-acclimated pea leaves (Liu et al., 2006). Some pharmacological and biochemical studies have demonstrated that PAC is an effective inhibitor of BA2H and is widely used as an inhibitor of SA biosynthesis (Ghai et al., 2002; Zhou and Zhong, 2011; Li et al., 2015). In the present study, it was found that the inhibition of BA2H activity by PAC effectively blocked SA accumulation. Similarly, exogenous PAC also limited the effects of exogenous SA by increasing H_2_O_2_, ${\text{O}}_{2}^{-}$ and MDA contents.

Our results showed that endogenous SA content and the PAL and BA2H enzyme activities were enhanced by exogenous SA, while they were inhibited by exogenous PAC under Al stress. PAL, a pivotal enzyme, is involved in defense mechanisms in plants. It is not only responsible for SA and phenolic acid synthesis, but also lignification of cell walls. Ou-yang et al. (2014) reported that PAL activities increased initially and then decreased with increasing Al concentration in cotyledons and radicles of *Jatropha curcas* seedlings. Al stress up-regulated SA-mediated *PAL* gene expression of potato during pathogen attack (Arasimowicz-Jelonek et al., 2014). BA2H catalyzes the final step, i.e., the conversion of benzoic acid to SA, which acts as a switch that directly controls the synthesis of SA (Jayakannan et al., 2015). The results that PAC treatment can aggravate Al-inhibited root elongation and reduce SA concentration in the root tips under Al stress, provide the evidence to explain the changes in the endogenous SA concentration caused by PAL and BA2H activities, which was associated with the Al toxicity syndrome in soybean (Liu et al., 2012).

NPR1 has been identified and characterized as a key component that functions downstream of SA in the signal transduction pathway (Spoel et al., 2003; Pieterse and Van Loon, 2004). In its inactive state, NPR1 resides in the cytoplasm as an oligomer bound by disulphide bonds. After induction of plant defense, SA activated a change of NPR1 from oligomeric to monomeric form, and then it was translocated to the nucleus and activates downstream target gene expression. *Arabidopsis snc1, sid2, nahG, npr1-1*, and *snc1/nahG* mutants that exhibited SA functions in response to Al stress were associated with the control of oxidative stress, but not of malate exudation (Guo et al., 2014). Kunihiro et al. (2011) documented that Al-induced *AtrbohD* expression and cell death were involved in NPR1-dependent SA signaling by using *npr1* mutant cells and NPR1 overexpressing cells. Using quantitative RT-PCR, we found that *GmNPR*1 expression increased continuously for 12 h after Al treatment; however, the overall transcript level was much higher in the first 6 h in the presence of SA and Al than that observed in the Al treatment alone. This may be due to different causes: (a) exogenous SA activates the expression of the *GmNPR1* gene under Al stress, and/or (b) SA serves as an endogenous secondary messenger to induce changes in ROS homeostasis, which resulted in the indirect activation of *GmNPR1* gene expression. It requires confirmation in future research whether the Al-resistance downstream genes can be regulated by *GmNPR1*.

Interestingly, the exogenous SA induced a transient increase in H_2_O_2_ at 6 h after Al stress, then the H_2_O_2_ content decreased with the elongation of treatment duration (9–12 h). This finding showed that SA-induced an H_2_O_2_ peak during the early stage of Al stress, suggesting that H_2_O_2_ might play an important role in regulating soybean tolerance to Al stress. A rapid H_2_O_2_ accumulation, also known as the oxidative burst, serves as a redox signal to regulate the plant tolerance to biotic and abiotic stresses (Hossain et al., 2015; Saxena et al., 2016). It was demonstrated that SA can cooperate with H_2_O_2_ to form a self-amplifying feedback loop in SA-mediated defense responses (Vlot et al., 2009; Miura and Tada, 2014). Chao et al. (2010) found that SA mediated H_2_O_2_ accumulation and helped rice plants withstand Cd toxicity. Dynamics of H_2_O_2_ content are triggered by SA under Al stress and are related to various antioxidant enzymes, such as, POD, SOD, APX, and CAT. The CAT serves as a key scavenging enzyme for H_2_O_2_ (Willekens et al., 1997; Lin et al., 2012; Mhamdi et al., 2012)_._ In the present study, SA decreased CAT activity under Al stress. However, SA and Al increased the activities of POD, SOD, and APX during the treatment. Therefore, the H_2_O_2_ content was decreased under Al stress. SA treatment alleviated Al-induced oxidative stress as shown by the decrease in the content of MDA and ${\text{O}}_{2}^{-}$. In rice, SA pretreatment enhanced Cd tolerance through elevated enzymatic and non-enzymatic antioxidants and the concentrations of glutathione and nonprotein sulfhydryl in roots and shoots, hence leading to alleviation of oxidative damage (Guo et al., 2009). Our result was different from the results in a study on *C. tora* plants subjected to Al stress, where SA induced changes in H_2_O_2_ levels by increasing the activity of POD, whereas the activities of CAT, APX, and GR remained unchanged (Wang et al., 2004). SA suppressed the Al-induced enhancement in SOD, GPX, and APX activities but alleviated the Al-induced reduction in CAT activity in rice (Pandey et al., 2013). In tomato, SA significantly reversed the Al-induced changes in the activities of SOD, POD, and CAT (Surapu et al., 2014). These discrepancies may be caused by the different research methods, the time course of measurements and plant species.

In conclusion, it is suggested that Al stress induced a remarkable increase in endogenous SA levels in soybean root tips via the PAL and BA2H enzymatic pathways. SA treatment regulated the expression of the *GmNPR1* gene under Al stress. SA induced H_2_O_2_, which functions as a signal and activates antioxidant systems to remove the excess H_2_O_2_; therefore, oxidative damage trigged by Al stress was partly alleviated.

## Author contributions

XZ, XL, and ZY conceived and designed the study; NL, FS, and JY carried out the experiments; NL and XZ analyzed the data; NL, XZ, and XL wrote the manuscript.

### Conflict of interest statement

The authors declare that the research was conducted in the absence of any commercial or financial relationships that could be construed as a potential conflict of interest.
